# The DIAMOND initiative: implementing collaborative care for depression in 75 primary care clinics

**DOI:** 10.1186/1748-5908-8-135

**Published:** 2013-11-16

**Authors:** Leif I Solberg, A Lauren Crain, Nancy Jaeckels, Kris A Ohnsorg, Karen L Margolis, Arne Beck, Robin R Whitebird, Rebecca C Rossom, Benjamin F Crabtree, Andrew H Van de Ven

**Affiliations:** 1 HealthPartners Institute for Education and Research, PO Box 1524, MS #21111R Minneapolis, USA; 2 Institute for Clinical Systems Improvement, 8009 34th Ave S, Suite 1200, Bloomington, MN 55425 USA; 3 Kaiser Permanente Colorado, Institute for Health Research, 10065 E Harvard Ave, Suite 300, Denver, CO 80231 USA; 4 Research Division, Robert Wood Johnson Medical School, New Brunswick, NJ, USA; 5 3-402 Carlson School of Management, University of Minnesota, 321 19th Ave S, Minneapolis, MN 55455 USA

**Keywords:** Depression, Implementation, Organizational change, Quality improvement, Collaborative care

## Abstract

**Background:**

The many randomized trials of the collaborative care model for improving depression in primary care have not described the implementation and maintenance of this model. This paper reports how and the degree to which collaborative care process changes were implemented and maintained for the 75 primary care clinics participating in the DIAMOND Initiative (Depression Improvement Across Minnesota–Offering a New Direction).

**Methods:**

Each clinic was trained to implement seven components of the model and participated in ongoing evaluation and facilitation activities. For this study, assessment of clinical process implementation was accomplished via completion of surveys by the physician leader and clinic manager of each clinic site at three points in time. The physician leader of each clinic completed a survey measure of the presence of various practice systems prior to and one and two years after implementation. Clinic managers also completed a survey of organizational readiness and the strategies used for implementation.

**Results:**

Survey response rates were 96% to 100%. The systems survey confirmed a very high degree of implementation (with large variation) of DIAMOND depression practice systems (mean of 24.4 ± 14.6%) present at baseline, 57.0 ± 21.0% at one year (P = <0.0001), and 55.9 ± 21.3% at two years. There was a similarly large increase (and variation) in the use of various quality improvement strategies for depression (mean of 29.6 ± 28.1% at baseline, 75.1 ± 22.3% at one year (P = <0.0001), and 74.6 ± 23.0% at two years.

**Conclusions:**

This study demonstrates that under the right circumstances, primary care clinics that are prepared to implement evidence-based care can do so if financial barriers are reduced, effective training and facilitation are provided, and the new design introduces the specific mental models, new care processes, and workers and expertise that are needed. Implementation was associated with a marked increase in the number of improvement strategies used, but actual care and outcomes data are needed to associate these changes with patient outcomes and patient-reported care.

## Background

Implementation and spread of proven improvements in care quality are widely recognized as major challenges for healthcare systems and clinics throughout the world. With the enormous diversity in practice setting, payment approach, and patient populations in the United States, it is particularly challenging to identify the factors and strategies that are associated with successful implementation. It is also difficult to determine the role of context in studies of implementation, since few publications provide enough information about the organizational context in which implementation takes place for readers to know whether lessons might apply in their organizations [1,2].

Depression care improvement provides an excellent opportunity to learn about implementation. While over 40 randomized controlled trials have provided a fairly clear picture of the collaborative care model changes needed for improvement in depression care, few of these changes have been implemented outside of research trials or were maintained after those trials ended, and there is rarely much information about either the implementation process or the organizational context in any publications [3-6]. Those studies that have addressed depression collaborative care implementation have either used interviews to understand barriers and facilitators to implementation or have relied on measures of clinician behavior for implementation [7-10]. None have actually measured the presence or functioning of practice systems designed to ensure systematic delivery of care model services. Since there is a wide gap between what we know works and what we usually do in depression care,, and since implementing the collaborative care model requires reliance on practice systems, it is particularly important to measure the extent to which they are in place.

**Figure 1:**
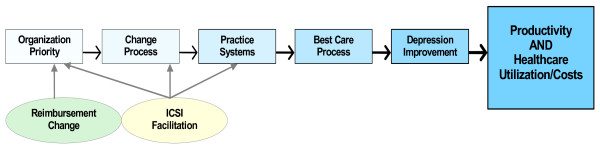
The implementation chain.

Both the successful implementation of evidence-based care and outcomes in the Veterans Administration care system and anecdotal accounts from investigators of other trials suggest that the lack of payment or resources for these proven services in most settings could be a significant barrier to implementation [11]. But if this barrier is reduced, what care changes can be implemented, and what organizational strategies and contextual factors are important? Our conceptual framework for implementation is based on the theory that the principal drivers of successful implementation are: first, organizational priority for the specific improvement goal; second, existing organizational capabilities to change; and finally, the types and extent of changes introduced as noted in the following diagram [12]. Changing reimbursement should incent an increase in priority, and the availability of facilitation should improve all three of these factors, leading to improved delivery of the desired care practices and improved patient outcomes and costs. Of course, there are many external and internal barriers and facilitators that might affect the likelihood that this chain of events will occur (see Figure 1).

We used this framework to evaluate a major statewide initiative in Minnesota to implement the collaborative care model for depression that was proven to be effective in the Improving Mood: Promoting Access to Collaborative Treatment (IMPACT) trial [13,14]. The Minnesota initiative, called DIAMOND (Depression Improvement Across Minnesota–Offering a New Direction) combined a new payment from all health plans in the state with externally facilitated implementation of evidence-based collaborative care management for adults with depression in 75 primary care clinics [15,16]. These clinics represented a wide diversity of ownership, location, and patient population, but overall were similar to primary care clinics in the state as a whole. We have previously demonstrated that practice systems for consistent care of depression were much less likely to exist in Minnesota medical groups than such systems for care of patients with diabetes or cardiovascular disease, so this Initiative provided an outstanding opportunity to learn about the real life issues of implementation [17].

## Methods

### General context

Minnesota has a large degree of consolidation of both its delivery and financing systems for medical care. There are six very large multi-specialty medical groups, and most of the remaining primary care physicians are in medium-sized groups, usually with multiple clinics within a single organization or medical group. While there are small clinics with one to four physicians, nearly all are part of larger groups, with very few independent small physician practices in primary care. Health plans are similarly consolidated, with three very large plans and four small ones.

Minnesota also has a high degree of collaboration among all these medical groups and plans, as well as with other stakeholders. One manifestation and contributor to this collaboration is the Institute for Clinical Systems Improvement (ICSI, http://www.icsi.org), a nationally prominent regional quality improvement collaborative with membership that includes medical groups containing 80% of the state’s physicians as well as most hospitals [18,19]. Its $4 million-a-year budget is largely covered by the five sponsoring health plans in addition to membership fees. Although ICSI began as a clinical guideline-creating organization, it soon added extensive guideline implementation efforts and later a variety of general quality improvement initiatives. All ICSI medical groups are assessed for organizational capacity for successful quality improvement before membership is granted, including an identified quality improvement coordinator and physician leader for quality, who are expected to guide the change management processes used at each of the group’s clinics. Upon admission to ICSI, each medical group receives on-going training in quality improvement. These medical groups have had considerable experience in implementing various quality improvement changes, although each has developed its own variations in the recommended structure and change processes to use.

An additional example of this collaborative environment is the presence of Minnesota Community Measurement (MN CM), another organization sponsored by all the Minnesota health plans for the purpose of standardized performance measurements that are publicly reported at http://www.mnhealthscores.org. After the DIAMOND Initiative began implementation, MN CM added compatible quality measures for depression care outcomes to its reporting system, which may have added another incentive to participate in the Initiative.

### DIAMOND initiative

Failing to see much improvement in depression care from either health plan disease management programs or its own quality improvement collaboratives, ICSI brought together the major stakeholders in 2006, including patients, clinicians, payers and employers. After reviewing evidence-based approaches to care delivery, a care approach was identified that followed ICSI’s treatment guidelines for depression [20] and that included all seven key components of an evidence-based collaborative care management program modeled after the approach used in the IMPACT study [3,4]:

1. Consistent use of a standardized tool (the PHQ9) for assessing and monitoring depression severity [21,22];

2. Systematic patient follow-up tracking and monitoring with a registry;

3. Treatment intensification for patients who did not improve;

4. Relapse prevention planning for those who go into remission;

5. A care manager in the practice to educate, monitor and coordinate care for patients in collaboration with the primary care physician;

6. Scheduled weekly psychiatric caseload review with the care manager in order to provide change recommendations to the primary care clinician for those not improving;

7. Monthly report of overall performance measures from each clinic for focused improvement attention, both by individual clinics and medical groups and the Initiative.

Since the group had identified the lack of a broadly-based supporting payment model as a significant barrier to implementation of this care model, it was clear that also needed to be part of the solution. Thus the DIAMOND Initiative was formed with a multi-stakeholder Steering Committee to guide piloting a new approach for the whole state. Further planning with multiple subcommittees led to encouraging all payers to provide a monthly bundled payment for those care model services that are not normally reimbursed. However, only clinics that successfully completed training by ICSI and demonstrated ability to follow the new care model would be eligible for this payment. In order to be eligible for this new payment, patients had to be age 18 or over and have a PHQ9 score of 10 or more.

In addition, each primary care clinic site had to include all adult primary care clinicians in the program, participate in a six-month training and implementation facilitation program managed by ICSI, provide ICSI with specified measurement information, and be certified by ICSI as having all of the above components in place. A total of 24 medical groups with 80 separate clinics and 553 full-time equivalent adult primary care physicians agreed to participate. Implementation was staggered, with groups of 10 to 26 clinics starting up every six months, from March 2008 to March 2010. The DIAMOND Steering Committee, with representatives from each local health plan, the Minnesota Department of Human Services, medical groups, DIAMOND study researchers, employers, and patients met monthly to monitor progress and make revisions with the help of several committees set up for specific issues (*e.g.*, measurement and reporting).

### Implementation

In order to facilitate implementation of the seven components described above in each clinic, ICSI staff developed the following approach:

1. **Recruitment of participants**

ICSI staff conducted a phone interview with each member medical group to assess interest in participating and readiness to take on this system redesign, as well as to identify which clinics would participate in which training sequence. The major factors used in both participation and timing for individual clinics were leadership interest, mutual assessment of readiness, and lack of competing priorities (*e.g.*, involvement in electronic medical record installation or leadership changes). Over the two and a half years of implementation, several clinics moved to later sequences because of lack of readiness, and others joined, presumably because they now felt more ready to take this on.

2. **Training**.

Participating clinics were required to include at least one physician, the care manager when hired, the care manager’s supervisor, and others who would be implementing this in four face-to-face sessions and two conference calls/webinars that covered each model component and system to be implemented. The first session used a gap analysis to allow teams to identify their greatest need areas, provided an overview of the model and the evidence-base behind it, and described how the workflow for delivering collaborative care differed from usual care. Each conference call/webinar and additional face to face session covered issues such as: PHQ-9 use, depression medication management and other clinical components for good clinical depression care, registry development and use, team building, role definition, and understanding of the new roles of care manager and consulting psychiatrist. In addition, a one and a half day training session was established for care managers just prior to implementation that focused on their role, workflow, and skill building for motivational interviewing, behavioral activation, and difficult cases.

3. **Training materials**.

These included a readiness assessment/gap analysis to identify where to focus attention and how to tailor training for individual needs. They also included checklists for implementation steps, example clinical workflows, evidence-based guidelines, slide sets covering curricula for each component, patient brochures and materials, and tools and scripts for each team member as well as a separate tool kit for the care manager. These training materials were used during training sessions and then given to the team in both paper and electronic formats.

4. **Registry**.

Arrangements were made with the AIMS Center at the University of Washington (Advancing Integrated Mental Health Solutions, http://uwaims.org/integrationroadmap/index.html) to provide participating clinics with use of its IMPACT-tested registry for enrolled patients unless a clinic could accomplish the same functions with its own automated registry system.

5. **Ongoing interactions after implementation**.

One month post-implementation, an all-team conference call was held to review progress on activating patients and to share care manager ideas and experiences. Three months post-implementation, a face-to-face “sharing session” was held to review implementation progress, barriers experienced in the first quarter and first measurement data. Monthly care manager network calls continued for the life of the Initiative for sharing and discussing new ideas, processes and tools that work, and challenges and frustrations. As each sequence of teams went through training and implementation, they then joined this existing networking group.

6. **Data submission for monitoring**.

A series of both process and outcome measures were developed by a measurement sub-committee, and each organization was trained for uploading the data needed for monthly submission and review of comparative performance. These measures were used for overall assessment of the Initiative, training modifications, individual site feedback for their own quality improvement work, and presentations. All data were shared without blinding with all participating clinic sites in order to facilitate collaborative learning and healthy competition.

7. **Other evaluation**.

At six and twelve months post-implementation, an assessment survey was sent electronically to gather information about implementation, sustainment and challenges. Also, site visits were conducted at six months by the ICSI DIAMOND team to each implementing clinic for both walk-through observations and additional questions. This information was used to help new groups understand the importance of each model component as well as for ongoing improvement by the existing groups.

### DIAMOND study

Working in partnership with the DIAMOND Initiative and its Steering Committee, a research study was proposed and funded by the US National Institute of Mental Health (NIMH) to evaluate the patient impacts and implementation actions of this Initiative [23]. The research design was based on a staggered implementation, multiple baseline approach, taking advantage of and mirroring the implementation schedule needed by the Initiative to make it feasible to train and facilitate change in all of these clinics [24,25]. The research design was also based on a conceptual framework for improving medical practice that the effects on depression care quality outcomes will depend on: first, the priority attached to the change by each clinic; second, the clinic’s change process capability; and third, the types of care process changes made [12]. This framework recognizes that the actual impact of these three key factors on outcomes is also potentially influenced by a variety of factors within and external to the clinic that could be barriers or facilitators for implementation [26].

Every clinic participating in the DIAMOND Initiative agreed to also participate in the DIAMOND Study. Two representatives from each clinic (the physician leader and the clinic manager) were invited to complete separate surveys that assessed priority, change process capability, and content of care processes changed. The process for recruiting respondents for these surveys involved first obtaining agreement from the medical group director to participate in the study. The director was then asked to identify the physician leader and clinic manager at each DIAMOND clinic and encourage them to complete the research surveys. The physician leader survey assessed care process changes and priority for change, while the clinic manager survey assessed change process capability and priority for change. Emails were then sent to these individuals at each of the 75 clinic sites that implemented the DIAMOND Initiative with a link to the appropriate electronic survey, prior to the start of the initiative and again one and two years after implementation. A weekly reminder email was sent if no response was received. Persistent non-respondents after three reminders (approximately 30%) then received phone calls from the project manager (KO) and principal investigator (LS) to verify that they had received the survey, to answer any questions, and to encourage response.

### Measures

#### Clinic and group characteristics

The information needed to describe participating clinics (see Table 1) was obtained from a survey of medical group and clinic managers that was conducted at the midpoint in the implementation. Incomplete or unreturned surveys were resolved by phone conversations with these people.

**Table 1:** Description of participating DIAMOND clinics (N = 75)

**Variable**	**N**	**% or Mean ± SD**	**Range**		
Location:					
• Metro Twin Cities	38	50.7%			
• Non-metro	37	49.3			
Ownership:					
• Health system	51	68.0%			
• Health plan	2	2.7			
• Physicians	21	28.0			
No. of adult PC MDs:		8.6 ± 7.8	1–39		
• 1–2	8	10.7%			
• 3–5	21	28.0			
• 6–10	33	44.0			
• >10	13	17.3			
Any in medical group:					
• Psychiatrists	37	49.3%			
• Mental health	38	50.7%			
• therapists					
No. of adult NP/PA:		2.1 ± 1.9	0–8		
• 0	16	21.3%			
• 1–2	32	42.7			
• >2	27	36.0			
No. of sites in medical grp:		15.6 ± 11.8	1–48		
• 1–2	4	5.3%			
• 3–5	16	21.3			
• 6–10	4	5.3			
• >10	50	66.7			
Patient insurance:					
• Commercial					
○ 0%–10%	2	2.7%			
○ 11%–25%	6	8.0%			
○ >25%	64	85.3%			
• Medicare					
○ 0%–10%	12	16.0%			
○ 11%–25%		35	46.7%		
○ >25%	25	33.3%			
• Medicaid					
○ 0%–10%		48	64.0%		
○ 11%–25%	20	26.7%			
○ >25%	4	5.3%			
• Uninsured					
○ 0%–10%	72	96.0%			
Participation reasons:					
• Improve dep’n	72	96%			
• Builds on prior work	65	87			
• ↑ care mgmt. capacity	63	84			
• ↑ mental health access	62	83			
• Support ICSI	50	67			
• Obtain reimbursement	26	35			

#### Care processes

The questionnaire, completed by the physician leader of each clinic site and used to measure the changes in practice systems, was a modification of one that had been developed by an expert panel for the National Committee on Quality Assurance (NCQA), called Physician Practice Connections (PPC). This questionnaire was originally designed to measure the presence of practice systems within the framework of the Chronic Care Model (CCM). The development process built on an extensive literature review, input from experts and key stakeholders, and reliability/validity testing with physician practices [27-29]. The PPC addresses five of the six domains of the CCM: health systems, delivery system redesign, clinical information systems, decision support, and self-management support [30]. It was subsequently modified by NCQA for use in its recognition program for patient-centered medical homes (as the PPC-PCMH) [31].

In order to modify the original PPC for this study, 53 (out of 98) individual items relevant to depression care or the CCM were selected that were not specific for various other chronic conditions. Many were modified, primarily by adding the term depression to the current wording of the question. Nine new items were then added to cover specific practice systems required for the DIAMOND initiative, as well as a previously tested question asking for the organizational priority for improving depression (on a scale from 1 to 10). This revised version, called the PPC for Research in Depression (PPC-RD), thus consisted of a total of 63 items and took about 10 minutes for physician leaders to complete. Since the intervention was going to start soon after the study was funded, there was no time for additional psychometric testing. Most items asked if specific practice systems were present and ‘work well’ or ‘need improvement’. Systems that were present but needed improvement received half credit (0.5) compared to those that worked well (1.0); no credit was given for systems that were not present (0).

We classified the items in the PPC-RD survey into three categories: DIAMOND (21 items specific to depression and consistent with the DIAMOND care management approach); non-DIAMOND depression (20 items relevant for depression but not specific for DIAMOND); and non-depression care (21 items relevant for any chronic condition). An overall measure of care process changes was calculated as the proportion (0% to 100%) of all care processes in the PPC-RD that were present and functioning well. Similarly, DIAMOND sub-scale was calculated from the 21 items that were specific to DIAMOND care management that was scored as the proportion of those items that were present (0% to 100%). A Depression-Specific sub-scale was comprised of the 21 DIAMOND and the 20 non-DIAMOND depression items; this scale did not include items unless they were directly relevant to practice systems for depression care (*e.g.*, items about immunization and a care manager for asthma were not included). A CCM scale was comprised of the 20 non-DIAMOND depression and the 21 non-depression CCM items; this scale did not include practice systems that were highly specific to the DIAMOND intervention (*e.g.*, measuring depression severity monthly, having a care manager for depression). In addition, the Depression-Specific and CCM scales were each subdivided into scores for each of the five domains of the CCM. Items from the original PPC, including those slightly modified to focus on depression, were assigned to the same CCM subscale they were assigned to in the original PPC. All scales were scored as the sum of system function ratings (*i.e.*, 0, 0.5, 1.0) included in a scale divided by the total number of systems rated on that scale, resulting in the proportion of systems that were present and functioning well. Thus, each scale represented the proportion of relevant systems that were present (while allowing half credit for systems present but not functioning well) as a percentage of perfect implementation (*i.e.*, 100%).

#### Change process capability

A second survey, completed by the manager of each clinic site, was used to measure organizational factors and improvement strategies used to implement the care system changes measured by the PPC-RD. This survey is called the Change Process Capability Questionnaire, or CPCQ, and is described in a 2008 publication [32]. It was created from items identified and prioritized by experienced clinic implementers in an iterative modified Delphi process, but has not been studied psychometrically [33]. The CPCQ scale has 30 items and 2 components [34]. The first component consists of 16 items that assess Organizational Factors and are rated on a five-point scale from Strongly Agree (5) to Strongly Disagree (1). Three subscales assess History of Change, Continuous Refinement, and Sustained Change. The Organizational Factors and its subscales are scored as the mean response to items in each scale, with higher values representing more favorable factors for successful change. The second component assesses strategies that have been used to implement improved depression care, and contains 14 items answered as Yes, Worked Well (scored 1); Yes, But Did Not Work Well (scored 0.5); or No, Not Used (scored 0). As with the PPC-RD, the Strategies scale was scored as the proportion of all items present and functioning well. The CPCQ also included a single question asking about the priority clinic leadership attached to this specific depression improvement effort relative to all other clinic initiatives on a scale of 1 to 10. Thus we obtained the separate perspectives on priority for depression improvement from both the physician and administrative leaders of each clinic.

### Analysis

Measures of central tendency and dispersion were calculated to describe clinic characteristics in a distribution-appropriate manner. Clinic-based PPC-RD and CPCQ scale scores were summarized at baseline and 1- and 2-year follow-ups as mean (M) and standard deviation (SD); changes were calculated as the raw difference between the 1- or 2-year and baseline scores. The significance of change in PPC-RD and CPCQ scores was assessed using paired t-tests.

This project was reviewed, approved and monitored by the HealthPartners institutional review board.

## Results

Seventy five (93.8%) of the 80 anticipated clinic sites completed and implemented the program. Of the 75 clinics implementing DIAMOND care after completing training, PPC-RD surveys were obtained from 74 physician leaders prior to implementation and from 73 leaders one year after implementation (response rate = 100% for at least 1 survey and 96% for both). Similarly, completed CPCQ surveys were obtained from 75 clinic managers before and 74 at one year after (response rate = 100% for at least 1 survey and 98.6% for both). These 75 clinics represented 20 separate medical groups with an average of 13 primary care sites ± 14 (median = 7). The response rates to the final round of surveys two years after implementation were 80% for the PPC (60 responses, 62 invitations) and 82.7% for the CPCQ (62 responses, 63 invitations). These reductions were due to the fact that by this time, 12 clinics had dropped out of the initiative and were not willing to continue completing research surveys, plus one clinic had merged so there was no comparable physician leader to provide PPC responses.

Table 1 describes the clinics participating in the Initiative and Study. They were distributed in the five sequences that began implementing every six months, beginning in March of 2008 as follows: Sequence #1 = 10, #2 = 20, #3 = 15, #4 = 12, and #5 = 18. Only 28% of the clinics were physician-owned, only 27% had fewer than 6 sites in their medical group, and the median number of adult primary care physicians in the clinic was 6. Psychiatrists were available within the medical group for 37 (49%) of these clinics to fill the DIAMOND role of care manager supervisor and consultant, but the other clinics had to arrange for this service from private practices in their area. Care managers had diverse backgrounds; 30% were certified medical assistants (CMAs), 40% were registered nurses (RNs), 15% were licensed practical nurses (LPNs), 2 sites used a nurse practitioner, 1 a psychologist, 1 a dietician, and 1 a physician.

The 12 clinics that dropped out were similar to those that remained in all characteristics except that they were more likely to have more than 2 nurse practitioners (83% vs. 27%, P = 0.001), and they had a different patient insurance type mix: 36% had less than 25% commercially insured patients vs. 7% for those that remained (P = 0.02). The leaving clinics had significantly higher PPC scores overall (46.3 vs. 36.4, P = 0.005) and for depression (35.9 vs. 22.2, P = 0.002) at baseline than those that stayed, but they didn’t rise as much to year 1 when there were no significant differences between these groups for any of the PPC scales or subscales. The same story was true for both CPCQ scales: higher at baseline for both Organizational Factors (1.2 vs. 0.8, P = 0.02) and Strategies (40.8 vs. 27.4, P = 0.03) but much less change over the next year. The Organizational Factors score for leavers fell during this time by −0.2 while clinics that stayed rose by the same amount, and the Strategies changes were +18.9 and +50.4 respectively. Finally, both the medical and manager leaders among the departing clinics provided similar priority ratings for improving depression at baseline to those in clinics that stayed, but at year 1, their ratings dropped by 1.6 and 1.3 points (10-point scale), while leaders in staying clinics rose by 1.1 and 0.4.

The presence of systems from the PPC-RD survey is shown in Table 2 for pre-implementation (baseline), one, and two years post-implementation. This documents that at baseline, both DIAMOND and general depression systems were at a low level (reporting only an average of 25% of the systems they could have), with a higher level (45%) for those general chronic care model systems largely unrelated to depression care. However, very large and significant increases occurred in the mean level of both DIAMOND and general depression systems and similar but smaller increases in mean chronic care model systems after implementation of DIAMOND. These mean increases reflected a normally distributed curve among the clinics (data not shown). For example, the mean overall score increased an absolute 24% at year 1, while 25% of the clinics increased by an absolute 37% or more, and 25% changed 12% or less (and 8% actually decreased over that year). Those clinics with the highest baseline PPC DIAMOND and general depression scores tended to have smaller increases in these scores at year 1. Clinics in earlier sequences tended to have more DIAMOND and depression-specific systems in place at baseline, but these differences between sequences were no longer present one year post-implementation. Overall, all PPC-RD changes at year 1 were positive and statistically significant at P = <0.0001, except CCM decision support (P = 0.004).

**Table 2:** PPC-RD Presence of systems at baseline and at one and two years post-implementation, mean ± SD (minimum, median, maximum)

**Scale**	Baseline*	1 Year post-Imp’n*	**2 Years post-Imp’n**
Number of clinics	74	73	58
Overall	38.0 ± 12.1	62.0 ± 17.0	62.1 ± 17.3
	(12.1, 36.3, 71.2)	(21.7, 62.0, 95.2)	(24.2, 60.5, 91.9)
DIAMOND	24.9 ± 16.3	60.6 ± 23.3	58.4 ± 22.8
	(0.0, 22.6, 71.4)	(11.9, 61.9, 100)	(4.8, 57.1, 97.6)
Depression:**	24.4 ± 14.6	57.0 ± 21.0	55.9 ± 21.3
	(0.0, 21.9, 71.9)	(18.5, 57.3,100)	(8.5, 54.3, 95.1)
Health systems	18.9 ± 20.4	50.1 ± 26.0	49.1 ± 27.6
	(0.0, 16.7, 91.7)	(0.0, 50.0, 100)	(0.0, 50.0, 100)
Delivery system redesign	22.5 ± 14.4	57.7 ± 21.3	57.1 ± 21.8
	(0.0, 18.7, 78.1)	(12.5, 56.3, 100)	(6.3, 56.3, 93.8)
Clinical information system	15.5 ± 20.3	43.2 ± 27.8	42.5 ± 24.6
	(0.0, 0.0, 66.7)	(0.0, 33.3, 100)	(0.0, 33.3, 100)
Decision support	36.2 ± 24.5	69.6 ± 24.6	68.3 ± 25.7
	(0.0, 40.0, 8.0)	(20.0, 70.0, 100)	(10.0, 70.0, 100)
Self-management support	27.4 ± 20.0	57.7 ± 25.8	56.0 ± 26.6
	(0.0, 27.2, 81.8)	(4.5, 59.1, 100)	(0.0, 54.5, 100)
Chronic Care Model:	44.7 ± 11.8	62.6 ± 15.4	64.0 ± 15.6
	(15.8, 42.6, 75.6)	(20.7, 62.2, 98.8)	(34.1, 64.6, 92.7)
Health systems	19.5 ± 21.1	49.0 ± 25.7	46.9 ± 27.4
	(0.0, 10.0, 90.0)	(0.0, 50.0, 100)	(0.0, 50.0, 100)
Delivery system redesign	33.6 ± 12.1	56.6 ± 17.8	57.8 ± 17.8
	(7.7, 30.7, 73.0)	(19.2, 53.8, 100)	(11.5, 55.8, 92.3)
Clinical information system	62.8 ± 17.2	71.5 ± 17.4	74.6 ± 20.5
	(6.2, 62.5, 73.0)	(31.3, 75.0, 100)	(31.3, 75.0, 100)
Decision support	69.4 ± 21.1	78.0 ± 20.1	79.7 ± 20.5
	(5.5, 72.2, 100)	(0.0, 77.8, 100)	(22.2, 83.3, 100)
Self-management support	28.9 ± 20.4	52.2 ± 24.2	54.4 ± 23.8
	(0.0, 25.0, 75.0)	(0.0, 50.0, 100)	(0.0, 50.0, 100)
Priority to improve depression	5.8 ± 2.3	6.5 ± 1.6	6.5 ± 1.6
	(1, 6, 10)	(3, 7, 10)	(2, 7, 10)

*Mean score for all PPC-RD scales is on a 0–100 ± SD, so the scores above represent the percent of maximum obtainable systems in each category or scale.

**These sub-scales don’t contain any items that do not address depression.

All PPC-RD changes at year 1 are positive and statistically significant at P = <0.0001, except CCM decision support = 0.004.

Priority to improve depression (self-rated on a 1 to 10 scale) increased at year 1 at P = 0.01.

None of the values at 2 years post-implementation are different from those at year 1.

These composite score changes do not tell the whole story of the changes in individual practice systems. The greatest change by far was in response to a question about presence of a system for depression care, which went from 93% No to 92% Yes at one year. The practice systems in the DIAMOND scale that had the largest increase after one year are identified in Table 3 along with the absolute difference between the mean scores at baseline and year 1.

**Table 3:** Individual practice systems with greatest changes from baseline to year 1

	**Yes, works well**	**Yes, needs improvement**
1. Non-physician responsible for guideline care:	+45%	+21%
2. Non-physician responsible for self-care education	+47%	+23%
3. System for depression treatment intensification	+40%	−14%
4. System for history of PHQ9 scores	+44%	−17%
5. System to assess depression treatment barriers	+43%	−14%
6. System to assess medication adherence	+43%	−8%
7. System to provide a relapse prevention plan	+41%	−2%
8. Systematic psychiatrist review	+43%	+2%

In contrast, systems showing very little change included those for identifying medications, general health risk factors, preventive services reminders, treatment guidelines (except for depression), and care management systems for care for diabetes, heart disease, or asthma. The clinics in the top quartile of change in their total PPC scores at year 1 increased these scores by an average of 46.0% (absolute), while those in the bottom quartile only increased their scores by 3.1% absolute, and one-third of these actually decreased their scores by 1 to 14 percentage points. There was no difference between top and bottom quartiles in the proportion of clinics that were not part of large medical groups (22% for each). Non-metro clinics were more likely to be represented in the top quartile (67% vs. 39%).

Out of the clinics still participating in the initiative with both year 1 and year 2 data, mean changes in PPC scores during the second year were very small. Thus there was no significant deterioration in the number of systems implemented at year 1, but no further improvement either, p >0.08 for all scale differences between year 1 and 2, and for the DIAMOND scale, p = 0.41.

Table 4 shows the scores for the CPCQ survey at baseline and at one and two years post-implementation. The organizational factor scales did not change during the first year, but the strategies used to improve depression care increased even more than the depression practice systems, suggesting that the change process for DIAMOND was as great an innovation among these clinics as the care processes and systems implemented. The only individual organizational factors that showed any change were strong agreement that the clinicians were interested in improving depression care (from 29.3% at baseline to 45.2% at one year), and strong agreement that the clinic uses quality improvement effectively (from 18.7% to 39.7%). Every one of the 14 strategies questions had large increases at year 1, but the greatest increases in strategies that were used and worked well were for making care more beneficial for the patient (+62%), making physician participation less work (+50%), and delegating care to non-physician staff (+53%). The strategies that had the smallest increases were reduction in the risk of negative results (28%), having goals and benchmark performance rates (38%), and reporting comparison performance rates (36%). The clinic managers completing this survey also gave a somewhat higher priority rating than physician leaders (see Table 2) to improving depression care (6.7 versus 5.8) at baseline, but unlike the physicians, they did not increase that priority at one year.

**Table 4:** CPCQ Quality improvement factors and strategies applied at baseline and change one and two years post-implementation, mean ± SD (minimum, median, maximum)

**Scale**	**Number of items**	**Score range**	**Baseline**	**1 Year post-implemen.**	**2 Years post-implemen.**
Number of clinics			75	74	62
Organizational factors, overall	16	−2 to +2	0.85 ± 0.59	0.96 ± 0.6	1.01 ± 0.6**
			(−1.3, 1.0, 1.8)	(−0.9, 1.1, 1.9)	(−0.9, 1.2, 1.8)
History of change	3	−2 to +2	0.98 ± 0.72	1.08 ± 0.8	1.22 ± 0.7**
			(−1.7, 1.0, 2.0)	(−1.0, 1.3, 2.0)	(−1.0, 1.3, 2.0)
Continuous refinement	3	−2 to +2	1.22 ± 0.76	1.37 ± 0.7	1.41 ± 0.7*
			(−2.0, 1.3, 2.0)	(−1.0, 1.3, 2.0)	(−1.3, 1.7, 2.0)
Sustain change	10	−2 to +2	0.70 ± 0.60	0.81 ± 0.6	0.83 ± 0.6*
			(−1.2, 0.8, 1.7)	(−0.8, 0.9, 1.9)	(−0.8, 0.9, 1.8)
Change strategies used in past year	14	0 to 100	29.6 ± 28.1	75.1 ± 22.3**	74.6 ± 0.2**
			(0.0, 26.8, 100)	(17.9, 82.1, 100)	(14.3, 82.1, 100)
Priority to improve depression	1	1 to 10	6.7 ± 1.7	6.9 ± 1.5	6.6 ± 1.9
			(2, 7, 9)	(3, 7, 10)	(1, 7, 10)

*P = <0.05 for change from baseline.

**P = <0.005 for change from baseline.

Much can be learned about implementation from failures, so the 12 clinics that dropped out were compared to those that remained. Those that left were similar in all characteristics except that they were more likely to have >2 nurse practitioners (83% vs. 27%, P = 0.001), and they had a much small proportion of their patients on commercial insurance (36% had <26% vs. 7%, P = 0.02). These departing clinics also had significantly higher PPC-RD scores at baseline (46.3% overall and 38.1% for DIAMOND vs. 36.4% and 22.4%, P = 0.004), but those differences disappeared at year 1. Their priority for improving depression was also higher when they started (7.2 vs. 5.6, P = 0.04), but it had dropped by 1.3 points at year 1 while that for clinics that stayed rose by 1.1 (P = 0.007).

At two years after implementation, the organizational factors had increased by enough from baseline (0.16) to be statistically significant (p < .005), while the use of improvement strategies did not increase further from the level at one year post-implementation.

Clinics in later sequences tended to have lower values for both PPC and CPCQ scales than those starting earlier. The assignment to a later sequence appears, as you might expect, to be related to a clinic not being prepared to implement DIAMOND on an earlier sequence. While there were no differences by sequence for PPC overall or the PPC Chronic Care Model subscale, sequence 5 clinics had lower scores than sequence 1 clinics for the more specific PPC DIAMOND and PPC depression subscales. Sequence 4 and 5 clinics also had lower priority scores than sequence 1 clinics. Similarly, there were no differences by sequence for the Organizational Factors scale, but clinics in sequences 2, 4 and 5 had lower scores on the Strategies scale than sequence 1 clinics.

## Discussion

These results show that this initiative was associated with large increases in the presence and functioning of depression systems at one year in most participating clinics. However, there was quite a large variation in this increase, with at least 18 clinics demonstrating no change or increasing less than 13%. At the same time, participating clinics reported use in that first year of many more strategies for improving depression care than they had reported at baseline. However, other organizational contextual factors had changed more slowly, becoming significant only two years after implementation began, suggesting that structural change is a developmental process that takes considerable time [35]. Of course, the final proof of successful implementation will be demonstration that the actual care received by patients and their outcomes changed along with this implementation. Analyses of these data are currently under way.

Nevertheless, these results on fidelity to the planned implementation processes and systems suggest that the DIAMOND Initiative approach to implementation was effective, although not with all participating clinics. Besides the rather wide variation in change in practice systems, 12 of the clinics originally implementing changes later dropped out. These clinics were much more likely to have a low proportion of commercially insured patients, so they would have had a much larger proportion of patients without the special reimbursement arranged by the commercial plans participating in this Initiative. More interesting, these departing clinics had higher scores for practice systems and for CPCQ readiness measures than clinics that remained through two years before they began implementation, but didn’t increase those scores as much at one year, while their priority ratings for improving depression actually fell. From the standpoint of predicting readiness to implement DIAMOND, these 12 clinics would have looked like excellent candidates, but something happened to prevent that, and they dropped out. ICSI staff working with these clinics believe that they dropped out for a variety of reasons, including insufficient patients with reimbursement coverage, lack of physician and leadership commitment, or not wanting to include or couldn’t find anyone to fill the psychiatry consulting role. Finally, some preferred to use an RN for case manager but didn’t find it cost-effective to do so.

The Initiative combined payment changes with an explicit proven set of care process changes and experienced training and facilitation among volunteer clinics with pre-existing quality improvement. There are undoubtedly other contextual and organizational factors that also contributed to this success. We are still at the stage in implementation research where well-documented descriptive case studies like this can provide valuable information for both researchers and implementation leaders. Other multi-method studies and analyses are ongoing to try to identify more specific facilitators, barriers, and predictive factors for successful implementation.

Wensing, Grol and others have suggested that adherence to guideline recommendations will be improved by designing implementation interventions that are tailored to local contextual barriers [36-38]. They categorized these barriers as care professionals, the organization of care, and social factors. In an earlier paper, we reported the results of qualitative interviews with 82 leaders of 41 medical groups in Minnesota, including most of those who later participated in the DIAMOND Initiative [39]. These leaders told us that there were three types of barriers to improving depression care in their organizations: external contextual problems–lack of reimbursement, scarce resources, and access to/communication with mental health specialists; individual resistant attitudes of both clinicians and patients; and internal care process problems–difficulty standardizing and measuring care for depression due to both organizational and condition complexity. This list is fairly similar to that proposed by Wensing *et al.*, and the DIAMOND Initiative directly addressed most of them by providing previously absent financial coverage, building in a link to mental health specialists, introducing a new care team member to facilitate standardization, and requiring measurement and whole clinic use of the new care system.

These findings are limited by our inability at present to test rigorously whether patient outcomes improved in parallel with these implementation measures, although these data will be forthcoming when other data are complete and fully analyzed. At that time, we will be able to test the relationship between implementation and outcomes at the level of individual clinics. The generalizability of these results is also limited by the somewhat atypical nature of primary care in Minnesota and the fairly high level of experience with quality improvement methods already present among these clinics as members of the ICSI collaborative for years. We also acknowledge the incomplete nature of psychometric evaluation of the surveys being reported and an inability to relate specific aspects of the intervention to specific barriers, since this was never done in planning to replicate the IMPACT approach. Finally, this subset of Minnesota clinics volunteered to participate in the DIAMOND Initiative on the basis of their leaders’ desire and their perceptions that they were ready to do so, at least during one of the five sequences over a two year period. Although the participating clinics were not randomized, the staggered implementation provides some evidence that the changes reported here were unlikely to be due to secular trends. What we report here are the results of a fairly intensive observational study of organizational change. We suspect, but cannot prove, that such change would not have been possible without the training and facilitation provided by ICSI.

This paper is intentionally limited to a description of the extent of implementation and maintenance of systems, as well as the presence of organizational readiness and change strategies used by participating clinics. We did not have room to provide our analyses of the predictive relationships between organizational characteristics, factors, and strategies and degree of implementation and do not yet have data on the extent to which the intervention changed care processes and patient outcomes. Those results will be presented in separate papers.

Nevertheless, this study demonstrates that under the right circumstances, primary care clinics that are prepared to implement evidence-based care can do so if financial barriers are reduced, effective training and facilitation are provided, and the new design introduces the specific mental models, new care processes, workers and expertise that are needed. That lesson might be usefully applied to many of the medical home demonstrations currently being implemented, often without those factors present.

## Competing interests

The authors declare that they have no competing interests.

## Authors’ contributions

LC conducted all the data management and statistical analyses for the paper, NJ provided all the Initiative description and assessments, KO managed all the data collection, KM helped with the assessment of practice systems measures, AB, RW, RR, BC, and AV provided critical expertise throughout the project and for this paper. Finally all authors provided important input on the developing article and read and approved the final manuscript.
